# ﻿Taxonomic position of holothurian *Eupentactafraudatrix* (Echinodermata, Holothuroidea)

**DOI:** 10.3897/zookeys.1197.117752

**Published:** 2024-04-18

**Authors:** Sergei V. Turanov, Alexey V. Smirnov, Yuri Ph. Kartavtsev

**Affiliations:** 1 A.V. Zhirmunsky National Scientific Center of Marine Biology, Russian Academy of Sciences, Vladivostok, Russia A.V. Zhirmunsky National Scientific Center of Marine Biology, Russian Academy of Sciences Vladivostok Russia; 2 Far Eastern State Technical Fisheries University, Vladivostok, Russia Far Eastern State Technical Fisheries University Vladivostok Russia; 3 Zoological Institute of Russian Academy of Sciences, St. Petersburg, Russia Zoological Institute of Russian Academy of Sciences St. Petersburg Russia

**Keywords:** 16S rRNA, COI, mitochondrial DNA, mtDNA, molecular phylogeny, *Sclerodactyla*, *Sclerothyone*, Sea of Japan, taxonomy

## Abstract

Samples of the holothurian *Eupentactafraudatrix* (Djakonov & Baranova in Djakonov, Baranova & Saveljeva, 1958) from the Sea of Japan were studied and the relationships of the genera *Eupentacta* and *Sclerodactyla*, as well as related taxa, were evaluated on the basis of phylogenetic analysis of the mitochondrial DNA COI and 16S rRNA genes. Using three methods, phylogenetic trees were constructed, and the degree of reliability of topological reconstructions was estimated by means of a nonparametric bootstrap test for the neighbor joining (NJ) and maximum likelihood (ML) techniques, as well as by a posteriori probability for Bayesian inference (BI) analysis. Genetic data confirm the validity of the assignment of *Cucumariafraudatrix* to the genus *Eupentacta* Deichmann, 1938. The study of sequences obtained from the holothurian specimens collected in Russian waters, near the city of Vladivostok, and determined by morphological characters clearly indicate that these specimens belong to the genus *Eupentacta* and are assigned as *E.fraudatrix* . The specimens from China in GenBank named as *Sclerodactylamultipes* and used in the present study, were likely misidentified, and after re-examination they may be assigned to the genus *Eupentacta*, either as *E.fraudatrix* or another taxon. Analyses of morphological characters of *S.multipes* unequivocally affirm that this species must be excluded from *Sclerodactyla* Ayres, 1851 and is provisionally assigned to the genus *Sclerothyone* Thandar, 1989 based on the external morphological characters and the body wall ossicles.

## ﻿Introduction

The holothurian *Eupentactafraudatrix* (Djakonov & Baranova in Djakonov, Baranova & Saveljeva, 1958) is currently used as a model species in numerous studies on biology, anatomy, histology, embryology, regeneration, biochemistry, chemistry of natural compounds, etc. A complete bibliography of studies conducted on *E.fraudatrix* up to 2015 was provided by Panina (2015).

The genus *Eupentacta* was erected by Deichmann (1938) to include the type species *Cucumariaquinquesemita* Selenka, 1867 and a new species that she described, *Eupentactapseudoquinquesemita* Deichmann, 1938. Panning (1949) established the family Sclerodactylidae and placed the genus *Eupentacta* in it. Unfortunately, he did not provide support for this decision, and subsequent researchers did not provide the dedicated considerations of the genus *Eupentacta* in their taxonomic studies. In the World Register of Marine Species (WoRMS 2024a, *Eupentacta* Deichmann, 1938; https://www.marinespecies.org/aphia.php?p=taxdetails&id=528610) , the genus *Eupentacta* is included in the family Sclerodactylidae with the following species assigned to it: *Eupentactaquinquesemita* (with *Cucumariachronhjelmi* Théel, 1886 as a synonym), *E.pseudoquinquesemita*, *E.fraudatrix*, and *E.exigua* (Ludwig, 1875)(WoRMS 2024a; *Eupentacta* Deichmann, 1938; https://www.marinespecies.org/aphia.php?p=taxdetails&id=528610).

*Eupentactafraudatrix* is very common in bays of Primorsky Krai, southwestern Sakhalin, and in the southern Kuril Islands at depths of 0–40 m, but mostly at 0–10 m. Morphology, anatomy, skeletal elements, and distribution of this species were described by Djakonov et al. (1958), Baranova (1971), Stukova and Levin (1990), Levin and Bekova (2005), and Panina (2015). This species was initially described in *Cucumaria* de Blainville, 1830 (Djakonov et al. 1958). Baranova (1979) placed this species in *Eupentacta* without providing evidence to support her viewpoint, but this placement was accepted by subsequent researchers without comment, and this species is currently considered to belong to that genus (WoRMS 2024a; https://www.marinespecies.org/aphia.php?p=taxdetails&id=528610).

The type species of *Eupentacta*, *E.quinquesemita* (Selenka, 1867), inhabits waters off the Pacific coast of North America from Kodiak Island and Baranof Island (Alaska) to southern California and Mexico at depths from 0 to 55 m. *Cucumariachronhjelmi*, which was described from Vancouver Island, was synonymized to *E.quinquesemita* by Deichmann (1938: 110). Lambert (1997) gave information on the biology of *E.quinquesemita* and the similar species *E.pseudoquinquesemita* and provided a list of publications including these species as model organisms off the west coast of Canada.

*Eupentactapseudoquinquesemita* has been found near the Commander and Aleutian Islands and along the North American Pacific coast from Kodiak Island to Puget Sound at depths from 0 to 228 m. A brief description of its morphology and skeletal elements was made available by Deichmann (1938) and Lambert (1997).

*Sclerodactyla* Ayres, 1851 had been considered a synonym of *Thyone* Oken, 1815 for many years. Panning (1949) restored it as an independent genus and designated it as the type genus of the family Sclerodactylidae, which he established. The type species of *Sclerodactyla* is *Holothuriabriareus* Lesueur, 1824 by original designation. Panning (1949) also placed *Cucumarialongipeda* Semper, 1867 (now *Phyllophorellalongipeda*, family Phyllophoridae) and *Cucumariamultipes* Théel, 1886 in *Sclerodactyla*, but without discussion. Since Panning (1949), *Sclerodactyla* has not been the subject of dedicated taxonomic research. In WoRMS (2024chttps://www.marinespecies.org/aphia.php?p=taxdetails&id=158531) , two species are assigned to *Sclerodactyla*: *Sclerodactylabriareus* (Lesueur, 1824) and *S.multipes* (Théel, 1886).

*Sclerodactylabriareus* lives in waters off the North American Atlantic coast from Nova Scotia to Florida, and in the Gulf of Mexico and off Venezuela. The species has been recorded from habitats at depths from 0 to 183 m (Hendler et al. 1995). The external morphology, anatomy, and structure of skeletal elements were described by Clark (1902), Coe (1912), Deichmann (1930), and Hendler et al. (1995). Information on this species’ biology and a brief overview of studies on *S.briareus* and its utility for biological research are provided by Hendler et al. (1995).

*Sclerodactylamultipes* was described from a single, fragmented specimen from Yokohama, Japan (Théel 1886). Later, specimens identified as *Cucumariamultipes* (= *Sclerodactylamultipes*) were collected off the Japanese coast, including at Yokohama and along the Sea of Okhotsk coast of Hokkaido Island (Mitsukuri 1912), in the Yellow Sea in Chinese waters, Chefoo Harbour (Chang 1948), and in the Korea Strait (Rho and Shin 1984; Shin and Rho 1996). These articles provide descriptions and drawings of the external morphology, the calcareous ring, ossicles from the body wall, introvert, podia, and tentacles.

Because the taxonomic position of *E.fraudatrix* has not been well established, we performed additional research on this species. In the last decade, molecular genetics methods have been regularly used taxonomy, including in the class Holothuroidea (e.g. Arndt et al. 1996; Miller et al. 2017). To clarify the systematic position of *E.fraudatrix*, we analyzed ribosomal RNA (rRNA) sequences in the 16S rRNA gene region and mitochondrial DNA (mtDNA) in the cytochrome *c* oxidase (COI) gene. We also used data from GenBank for a comparative analysis (https://www.ncbi.nlm.nih.gov/) with subsequent verification using the MIDORI server (Leray et al. 2018). 16S rRNA and COImtDNA sequences were obtained from *E.quinquesemita*, the type species of the genus *Eupentacta*, and *E.pseudoquinquesemita*. Sequence data were also collected for the following species of *Sclerodactyla*, the type genus of the family Sclerodactylidae: *S.briareus*, the type species of the genus, and *S.multipes*. Unfortunately, sequences of Chinese specimens treated as *S.multipes* in GenBank and used in the current study have not been published, nor is their identity supported by morphological data; obviously they cannot be attributed with certainty to *S.multipes*. In other words, the possibility of misnamed specimens (as sometimes happens with GenBank records) cannot be ruled out. Despite this, we use the name “*S.multipes*” to refer to these specimens, as they appear in GenBank.

## ﻿Materials and methods

Three individuals of *Eupentactafraudatrix* were collected from a depth of 1–4 m in Patrokl Cove, Peter the Great Bay, Sea of Japan (43.1619°N, 131.9164°E) in June 2021. From these individuals, tissue specimens were fixed in 95% ethyl alcohol. DNA was extracted from muscle tissue using a K-Sorb kit (Syntol LLC, Moscow). A PCR was performed using the primers 16Sar/16Sbr (Palumbi 1996) and LCO1490/HC02198 (Folmer et al. 1994), respectively, with a 20 µL cocktail including 10 µL AmpliTaq Gold 360 Master Mix (ThermoFisher Scientific, USA), 0.5 µL of each (forward and reverse) primer (10 µM), 0.16 µL bovine serum albumin, 1 ng DNA, and deionized water for the remaining volume. The PCR algorithm for both fragments consisted of preheating at 94 °C for 2 min and then 35 cycles with denaturation at 94 °C for 30 s, annealing at 42 °C for 40 s, and elongation at 72 °C for 1 min; the final stage of elongation lasted 10 min. Amplicons were verified using electrophoresis in an 1% agarose gel (Helicon, Russia; https://www.helicon.ru/), visualized with ethidium bromide under transmitted UV light. The alcohol-purified amplicons were used for forward and reverse stepping sequencing with appropriate primers (see above) and a BrightDye™ Terminator Cycle Sequencing Kit v. 3.1 (NimaGen, The Netherlands). Capillary electrophoresis was performed on an ABI Prism 3500 DNA sequencer. The obtained chromatograms were edited in Geneious (Kearse et al. 2012). The sequences were deposited in GenBank (Benson et al. 2018) under accession nos. OR288149–OR288151 (COI) and OR289514–OR289515 (16S rRNA). Original specimens of fixed animals are at the NSCMB FEB RAS in the personal collection of S.V. Turanov.

**Figure 1.**
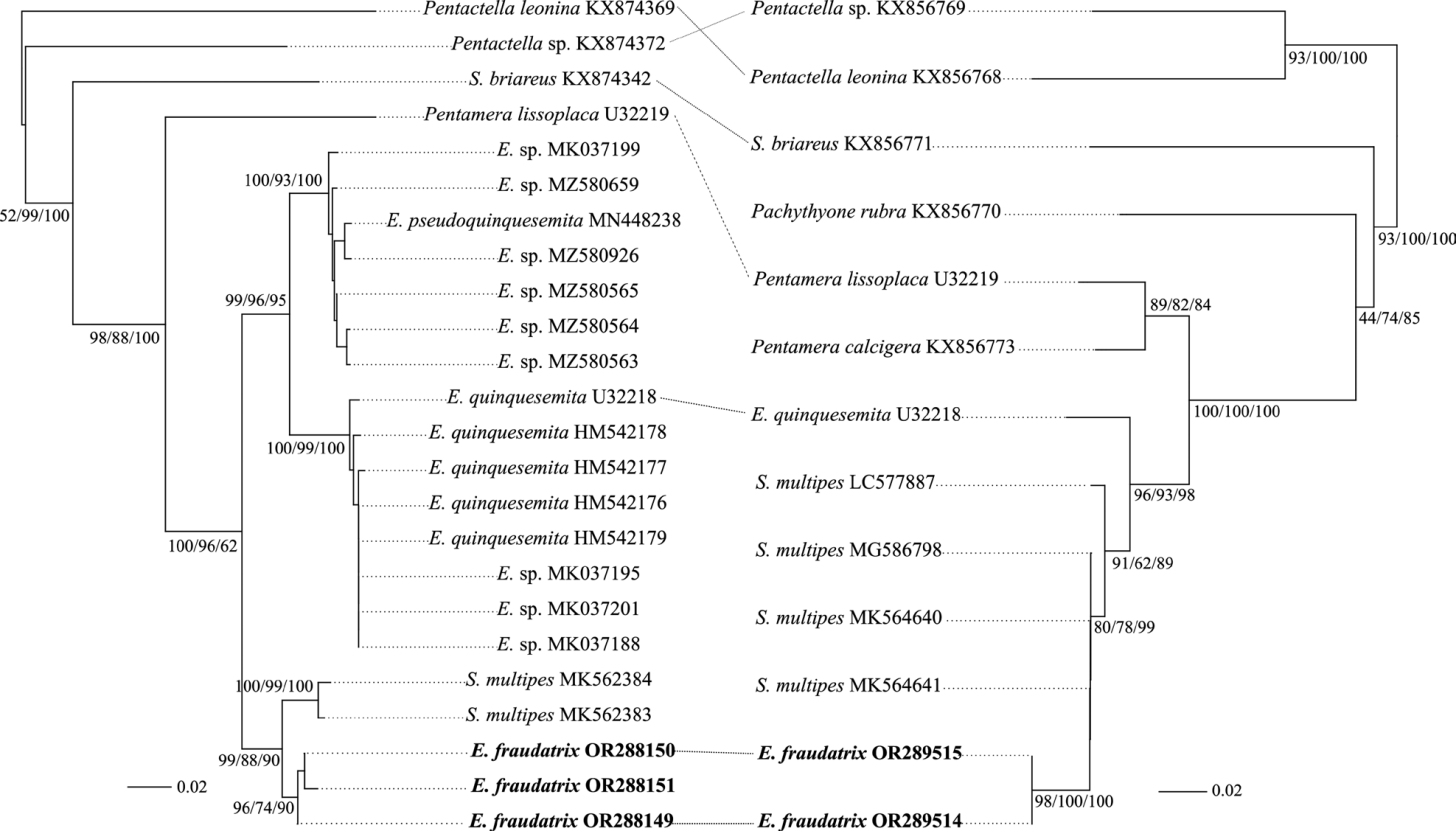
Co-phylogram plot showing relationships of the genera *Eupentacta* and *Sclerodactyla*, with related holothurians, as inferred from a phylogenetic analysis of the COI (left) and 16S rRNA (right) gene sequences. The trees were rooted at a midpoint. The numerals at the nodes are nonparametric bootstrap test (NJ, ML) and *a posteriori* probability (BI, %) values (the order is NJ/ML/BI). The dotted line connects the identifiers of the sequences from the same specimen. The sequences obtained in the present study are indicated with bold letters. The scale on the left and right bottom shows the relative length of branches in two gene trees.

We accessed 21 COImtDNA and 11 16S rRNA sequences from GenBank for comparison. Alignment was carried out separately for each marker in the MEGA 7 software package (Kumar et al. 2016) by the Muscle algorithm (Edgar 2004). Estimation of genetic distances, *p*-distances, was also performed in MEGA 7 (Nei and Kumar 2000). External indels were encoded as missing data (“?”). Selection of models for nucleotide substitutions and phylogenetic analysis of sequences by the neighbor-joining (NJ) and maximum-likelihood (ML) techniques with evaluation of the stability of topologies by the nonparametric bootstrap test was carried out using a set of respective functions in the phangorn (Schliep 2011) and ape packages (Paradis et al. 2004) of the R environment (R Core Team 2021). Bayesian-inference (BI) phylogeny was inferred in the MrBayes 3.2.7 software (Ronquist et al. 2012). A complex simultaneous selection of an optimal model for nucleotide substitutions implemented in MrBayes and gene partitions that that were taken into account (COI sequences) and were not taken into account (16S rRNA sequences) was made on the basis of AIC value in the PartitionFinder 2.0 software (Guindon et al. 2010; Lanfear et al. 2012, 2014). The search for tree topology and marginal values of *a posteriori* probability was performed by running four Markov chains for *n* = 106 generations. The rate of sampling topologies and parameters by the metropolis algorithm was 1 per 100 generations. The first 25% of the trees corresponding to the burn-in step were discarded as suboptimal. A consensus tree was constructed based on the remaining *n* = 15,002 topologies. The convergence indices (ESS, PSRF) indicated a sufficient sample for all parameters. Combined visualization of the topologies was obtained by the *cophylo* function in the phytools package (Revell 2023). The NJ topology comprises the basic tree for comparison and two others used for illustrative needs (Fig. 1).

## ﻿Results and discussion

The length of the COI sequences matrix after alignment was 888 base pairs (bp). Of these, 267 sites were variable, including 162 parsimony informative and 103 singleton sites. The 16S rRNA sequence matrix after alignment consisted of 569 bp, of which 186 were variable, including 97 parsimony informative and 88 singleton ones. In the COI phylogenetic tree (Fig. 1), a branch with significant support was formed that combined two deeply divergent groups consisting of the sequences of *Eupentactapseudoquinquesemita* and *E.quinquesemita* (group 1, with high support scores of 99/96/99% for the three techniques used, consequently), and also *S.multipes* and *E.fraudatrix* (group 2, with support scores of 99/88/90%). *Eupentactapseudoquinquesemita* and *E.quinquesemita* were well-supported sisters, as were *S.multipes* and *E.fraudatrix*. The divergence between members of these two groups have a *p*-*distance* of 0.06–0.07, and 0.05 between *E.pseudoquinquesemita*–*E.quinquesemita* and 0.03 between *S.multipes*–*E.fraudatrix*. Intraspecific *p*-distances were all ≤0.01. *Sclerodactyla* was paraphyletic. However, in view of the possible mislabeling of five specimens from China, we have to raise a question about the presence of a new taxon in the area and will send a note to GenBank with the suggestion of relabeling these five specimens. The rank of this taxon is subspecies–species, as we clarify below.

The 16S rRNA-based tree showed less comparative material, but the relationships of the genera *Eupentacta* and *Sclerodactyla*, similarly, is paraphyletic and certain tree topologies are unresolved. The main branch is well supported, but represented by a large number of taxa, including *Pentamera* Ayres, 1852, whose species diverged from other representatives with the *p*-distance of 0.08–0.11 (mean 0.09 ± 0.01). The interspecific *p*-distance in this genus was 0.05. In the *Eupentacta*–*Sclerodactyla* branch, which is also highly supported, the closest branch comprised only of *E.fraudatrix* sequences. *Eupentactaquinquesemita* took a basal position here. The interspecific divergence ranged from 0.03 (*E.fraudatrix*–*S.multipes*) to 0.07 (*E.fraudatrix*–*E.quinquesemita*). The intraspecific variability was as above ≤0.01. The order of species, depending on distance from the main branch, was as follows: *Pachythyonerubra* (Clark, 1901) (divergence with respect to *E.fraudatrix*, 0.21) and *Sclerodactylabriareus* (0.22). Representatives of the genus *Pentactella* Verrill, 1876 formed a clear outgroup in this topology.

In this paper, we combined information on 20 specimens for COI and seven specimens for 16S rRNA from the genus *Eupentacta* (Fig. 1). Five specimens from one area of China (unfortunately, no precise information was given on the sample location by the submitters of sequences to GenBank, but provisionally the samples were collected near Qingdao City) are obviously not a comprehensive sample. We are going to expand our research in the future, but for this paper, the present samples appear suitable for a valid conclusion. Thus, the divergence between specimens from Russia and China is close to 3%, as it is given above. However, this amount of divergence cannot be explained by the intraspecies variability, e.g., even our own dataset presented above and the one comprised of COI and 16S rRNA sequences provide supportive information. The divergence between members of two groups of *S.multipes* and *E.fraudatrix* in the *p*-distance value was 0.06–0.07 (6–7%) with a mean of 0.065 ± 0.005. The divergence within the groups was 0.05 (*E.pseudoquinquesemita*–*E.quinquesemita*) and 0.03 (*S.multipes*–*E.fraudatrix*). The intraspecific *p*-distances in all the above-listed species were not greater than 0.01 (1%). Looking at a comprehensive review of over 20,000 invertebrate and vertebrate specimens, the score of genetic distances estimated at different taxon levels could explain the matter (e.g. Kartavtsev 2011, 2013). The distance data revealed increasing levels of genetic divergence of the sequences of the two genes, cytochrome b (Cyt-b) and COI, in the five groups compared: (i) populations within a species; (ii) subspecies, semi-species, or/and sibling species; (iii) species within a genus; (iv) species from different genera within a family; and (v) species from separate families within an order. The mean unweighted scores of *p*-distances (%) for these five groups for Cyt-b were as follows: (i) 1.38 ± 0.30; (ii) 5.10 ± 0.91; (iii) 10.31 ± 0.93; (iv) 17.86 ± 1.36; and (v) 26.36 ± 3.88, respectively; and for COI, the scores were the following: (i) 0.89 ± 0.16; (ii) 3.78 ± 1.18; (iii) 11.06 ± 0.53; (iv) 16.60 ± 0.69; and (v) 20.57 ± 0.40. Evidently, the intraspecies level for these two gene markers is approximately 1%, i.e., that is the exact value we had in our *multipes*” and “*fraudatrix*” data for the two analyzed genes.

We also must consider whether *S.multipes* from China is a separate taxon. The topology of the gene trees for COI and 16S rRNA may be even more important, and this allows us to place the Chinese samples jointly with other *Eupentacta* species, i.e., the topology in that part of the whole tree combines “*multipes*” and “*fraudatrix”.* The two trees show high support for the integrity of each of the two datasets by the three techniques of tree building (Fig. 1; 99/88/90% for COI and 80/78/99% for 16S rRNA). Thus, molecular genetics alone give good support to the Chinese samples as an independent taxon at the subspecies/semi-species level and place it, with the reservations made in the Introduction, definitely near the genus *Eupentacta*. DNA barcoding considers a 2–4% difference the threshold for the intraspecies/species discrimination (Ward 2009; Turanov and Kartavtsev 2014).

A comparison of the morphological traits of *S.briareus* and *S.multipes* clearly indicates that these species do not belong to the same genus. In *S.briareus*, tube feet are located all over the body, while in *S.multipes* they are bounded only by radii, as in *Eupentacta*. In *S.briareus*, the radial and interradial plates of the calcareous ring are connected for two-thirds of their length (Clark 1902: pl. 13, fig. 95; Panning 1949: 459); in *S.multipes*, the structure of the calcareous ring in the holotype is unknown, but in the specimens that were subsequently identified as *S.multipes*, the calcareous ring plates are connected only in the lower part (Chang 1948: 77, fig. 20j; Shin and Rho 1996: 541, fig. 136). The close similarity of Chinese sequences to *E.fraudatrix* sequences raises doubts that Chinese researchers correctly identified the studied specimens, since the structure and composition of the ossicles vary greatly between *E.fraudatrix* and *S.multipes*. It is possible that these workers were not dealing with *S.multipes*, but instead with а close relative of *E.fraudatrix*, although neither *E.fraudatrix* nor forms close to this species had previously been recorded off the Chinese coast. The taxonomic morphological characters of *S.briareus* and *S.multipes* clearly indicate that *S.multipes* must be excluded from *Sclerodactyla*. *Sclerodactylamultipes* should provisionally be placed in the genus *Sclerothyone* Thandar, 1989 based on the structure of the ossicles of the body wall. This placement is supported by the fact that the closely related *Havelockianozawai* (Mitsukuri, 1912), described from Japanese waters and considered a possible synonym of *Sclerodactylamultipes* by Chang (1948: 76), was recently placed in *Sclerothyone* by Thandar (2021: 509–511).

## ﻿Conclusions

The obtained molecular genetics data confirm the assignment of *Cucumariafraudatrix* from Russian waters to the genus *Eupentacta*. *Sclerodactylamultipes* unequivocally cannot remain in the genus *Sclerodactyla* and, based on morphological characters, should provisionally be placed in *Sclerothyone*. In the diagnosis of *Eupentacta*, the molecular data and morphology of the calcareous ring structure are considered. The diagnosis is given below.

The position of the genus *Eupentacta* in the system of the order Dendrochirotida remains poorly resolved. Based on the calcareous ring structure, *Eupentacta* does not fit well in the family Sclerodactylidae. In the description of *Eupentacta* (Deichmann 1938: 110), it was noted that it is intermediate in position between the genera *Pentamera* (family Thyonidae) and *Pentacta* Goldfuss, 1820 (family Cucumariidae). Our analysis of molecular data indicates the proximity of *Eupentacta* to *Pentamera* (Fig. 1). Our study supports the need for a revision of the order Dendrochirotida, based on both molecular genetics and morpho-anatomical data collected by modern methods. Until the families Sclerodactylidae, Sclerothyonidae, and Thyonidae are revised, we temporally leave *Eupentacta* in the Sclerodactylidae. Below is an updated diagnosis of the genus *Eupentacta*.

**Figure 2.**
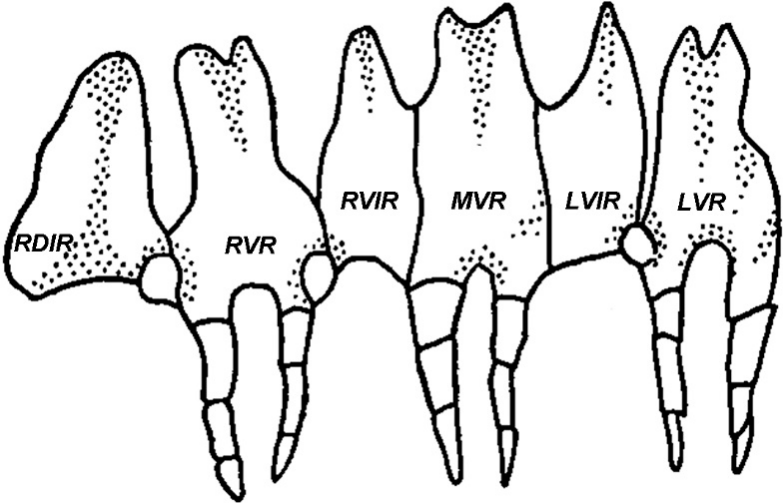
Part of the calcareous ring of *Eupentactafraudatrix*. RDIR, right dorsal interradial plate; RVR, right ventral radial plate; RVIR, right ventral interradial plate; MVR, medioventral radial plate; LVIR, left ventral interradial plate; LVR, left ventral radial plate (from Baranova 1971).

**Figure 3.**
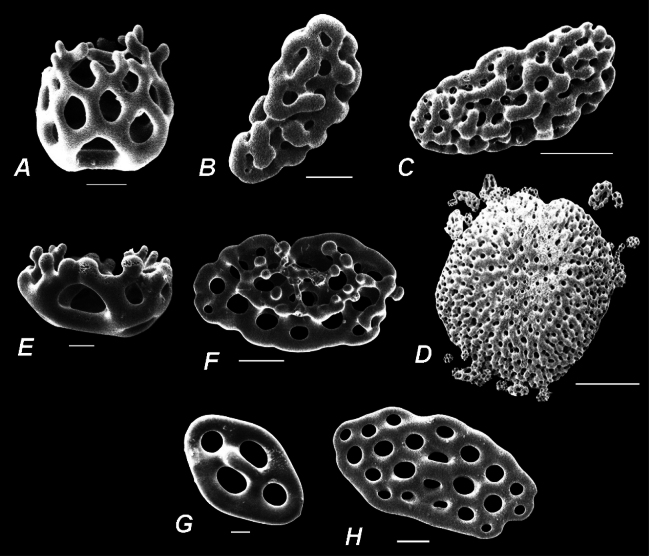
SEM photomicrographs of the body wall ossicles of *Eupentacta* species **A–D***E.quinquesemita*, Mendocino, California (holotype, Museum of Comparative Zoology at Harvard University) **A** basket **B** knobbed plate **C** fenestrated hollow ellipsoid **D** very large scale-like multilayer plate **E, F***E.pseudoquinquesemita*, Kodiak Island, Shelikof Strait, Uyak Bay, Heard of Larson’s Inlet, Alaska (paralectotype, United States National Museum E2288) **E** basket **F** irregular plate with tubercles rising above **G, H***E.fraudatrix*, Peter the Great Bay, Vostok Bay, Russia **G** plate with oval disk with four holes and a handle-like arch between the two holes in the longitudinal axis (underdeveloped table with modified 2-pillared spire?) **H** plate with small elevation in the center. Scale bars: 10 µm (**A, B, E, G**); 30 µm (**F, H**); 100 µm (**C, D**).

Figs 2, 3 (a drawing and an SEM photomicrograph) illustrate the key morphological characters of the genus *Eupentacta*.

### ﻿Emended, updated diagnosis

#### ﻿Genus *Eupentacta* Deichmann, 1930

Medium-sized holothurians, with cylindrical body and rounded posterior end. Tube feet confined to ambulacra; 10 tentacles, two ventral tentacles smaller than others; anus surrounded by five small anal papillae. Radial plates of calcareous ring with fairly short posterior processes smaller in length than plate height; posterior processes consisting of 2–3 pieces; anterior part of radial plates narrowed toward anterior margin; small notch present on anterior margin; interradial plates triangular, pointed anteriorly, without posterior processes; medioventral radial plate and two adjacent interradial plates fused together in the lower and middle parts for approximately ⅔ of plate height into single plate; other radial and interradial plates separate and articulate with each other only in their lower part. Body wall ossicles: in outer layer of body wall in form of baskets; in deeper layers of body wall different ossicles specific to each species present: large knobbed plates, fenestrated hollow ellipsoids and very large scale-like multilayer plates (in *E.quinquesemita*); irregular plates with tubercles rising above (in *E.pseudoquinquesemita*); plates usually with oval disk with four or sometimes more holes and a handle-like arch between the two holes in the longitudinal axis in the middle layer (underdeveloped tables with modified 2-pillared spire?), and different plates with 4–6 and more holes up to large massive plates with numerous holes with small elevation in the center in the deep layer (in *E.fraudatrix*). Tube feet ossicles supporting tables with elongated narrow base and small column or with reduced column in form of bridge, and large, well-developed, rounded end plate. Tentacle ossicles elongated rods with small holes.

**Type species.***Cucumariaquinquesemita* Selenka, 1867.

**Other species included.***Eupentactapseudoquinquesemita* Deichmann, 1938; *Cucumariafraudatrix* Djakonov & Baranova in Djakonov, Baranova & Saveljeva, 1958, and in question *Cucumariaexigua* Ludwig, 1875.

*Eupentacta* differs from other genera of Sclerodactylidae and Thyonidae in the structure of the calcareous ring: the medioventral radial plate is fused with the adjacent interradial plates. *Eupentacta* also differs from *Sclerodactyla* Ayres, 1851 and *Pachythyone* Deichmann, 1941 (Sclerodactylidae) in having the podia restricted to radia, scattered over the whole body in *Sclerodactyla*, settled along the radii and numerous in the interradii in *Pachythyone*, and strictly located along the radii in *Eupentacta*; from the genus *Sclerothyone* (Sclerothyonidae) it differs by the presence of baskets in the surface layer of the body wall, and the absence of tables with well-developed 2-pillared spires in the body wall. It differs from *Pentamera* Ayres, 1852 (Thyonidae) by body shape: curved upwards and gradually tapering posteriorly in *Pentamera* and cylindrical with rounded posterior end in *Eupentacta*.

## ﻿Additional information

### Author ORCIDs

Sergei V. Turanov https://orcid.org/0000-0003-4829-9729

Alexey V. Smirnov https://orcid.org/0000-0002-7350-9276

Yuri Ph. Kartavtsev https://orcid.org/0000-0002-0436-4763

### Data availability

The gene sequence data of this study are openly available in GenBank of NCBI at (https://www.ncbi.nlm.nih.gov/) under the accession nos.: OR288149–OR288151 (COI) and OR289514-OR289515 (16S rRNA). Other options could be requested from the authors.
